# 10,11-Dehydrocurvularin attenuates LPS-induced neuroinflammation in BV2 cells by inhibiting the TLR2/MyD88/NLRP3 signaling pathway

**DOI:** 10.1016/j.bbrep.2026.102718

**Published:** 2026-07-18

**Authors:** Chenggang Tian, Jian Sun, Jinting Wang, Yu Wang, Peifeng Wan, Guangqiang Sun, Yanling Mu, Meiyu Geng, Baofu Xu, Yu Zhang

**Affiliations:** a Shandong First Medical University & Shandong Academy of Medical Science, Jinan, Shandong, 250117, China; b Shandong Laboratory of Yantai Drug Discovery, Bohai Rim Advanced Research Institute for Drug Discovery, Yantai, Shandong, 264117, China; c State Key Laboratory of Drug Research, Shanghai Institute of Materia Medica, Chinese Academy of Sciences, Shanghai, 201203, China

**Keywords:** 10,11-Dehydrocurvularin, Neuroinflammation, TLR2, BV2

## Abstract

Neuroinflammation, driven by microglial activation and Toll-like receptor 2 (TLR2) signaling, is a hallmark of neurodegenerative diseases. While 10,11-Dehydrocurvularin (DCV) is known for its anti-inflammatory properties, its specific effects on neuroinflammation remain poorly understood. This study evaluated DCV in lipopolysaccharide (LPS)-stimulated BV2 microglia. Nitric oxide (NO) was measured via the Griess assay, interleukin-1β (IL-1β) and IL-6 levels were quantified using ELISA or RT-qPCR, and the TLR2/MyD88/NLRP3 pathway was analyzed via Western blotting. To validate the target specificity, we employed a TLR2 agonist (Pam3CSK4), a pharmacological inhibitor (C29), and siRNA-mediated knockdown. Results showed that DCV concentration-dependently inhibited LPS-induced NO production (IC_50_ = 3.501 μM) and significantly reduced IL-1β and IL-6 at both transcriptional and translational levels. Mechanistically, DCV suppressed the LPS-induced upregulation of TLR2, MyD88, and activation of the NLRP3 inflammasome. Notably, the TLR2 agonist Pam3CSK4 reversed the inhibitory effects of DCV on downstream inflammatory mediators. Conversely, the anti-inflammatory effect of DCV was occluded by TLR2 inhibition; neither pharmacological inhibition (C29) nor genetic knockdown (siRNA) of TLR2 allowed DCV to further reduce NO levels, indicating that DCV acts through a TLR2-dependent pathway. Transcriptomics profiling further revealed that DCV modulated immune-related genes, enriching the cytokine-cytokine receptor interaction and chemokine signaling pathways. Collectively, these findings demonstrate that DCV exhibits potent anti-neuroinflammatory activity by suppressing the TLR2/MyD88/NLRP3 signaling pathway, suggesting its potential as a promising lead compound for neuroinflammation-associated neurodegenerative diseases.

## Highlights

• DCV inhibits LPS-induced neuroinflammation in BV2 microglial cells.
• DCV downregulates TLR2, MyD88, and NLRP3 protein levels.
• The TLR2 agonist Pam3CSK4 reverses the anti-inflammatory effects of DCV.
• TLR2 blockade abrogates DCV's anti-inflammatory synergy, confirming pathway dependence.
• Transcriptomics reveals DCV modulates immune and chemokine signaling pathways.

## Graphical abstract

**Figure:**
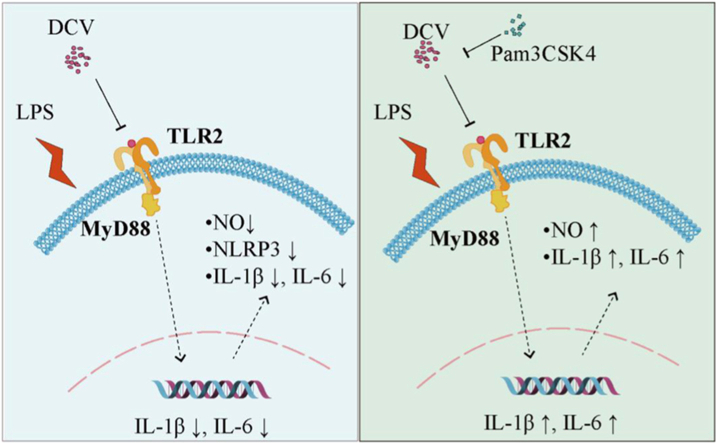

## 1 Introduction

Neurodegenerative diseases (NDDs), including Alzheimer's disease (AD), Parkinson's disease (PD), amyotrophic lateral sclerosis (ALS), and Huntington's disease (HD), constitute a clinically diverse group of disorders characterized by progressive neuronal loss. Although their etiologies vary widely, ranging from distinct protein aggregation cascades to specific genetic mutations, they share a critical pathological hallmark: chronic neuroinflammation [1]. For instance, elevated levels of cytotoxic T cells have been observed in the brains of tauopathy and AD mouse models [2]. Similarly, in patients with PD dementia, microglial activation within the amygdala correlates significantly with α-synuclein pathology and is accompanied by the infiltration of CD4^+^ T cells [3]. Identical neuroinflammatory processes have also been well-documented in stroke patients and corresponding rodent models [4,5]. Consequently, targeting the central nervous system (CNS) immune response, which culminates in the activation of resident microglia and astrocytes along with the infiltration of peripheral immune cells, has become a pivotal strategy in drug development. To date, several candidates have entered clinical evaluation. Minocycline (MINO), for example, recently completed a Phase III clinical trial for acute ischemic stroke, demonstrating significant functional benefits at 90 days alongside a favorable safety profile compared to placebo [6]. Semaglutide has shown promise in preclinical studies by promoting a microglial M1-to-M2 phenotypic transition, thereby inhibiting neuroinflammation, reducing hippocampal Aβ deposition, and improving cognitive impairment in 3xTg-AD mice [7]. However, while large-scale Phase III trials of semaglutide in early AD are ongoing, its primary cognitive endpoint (Clinical dementia rating scale-sum of boxes [CDR-SB]) was recently reported as unmet [8]. Despite these incremental advances, effective therapies capable of targeting the upstream drivers of neuroinflammation remain elusive.

Microglia, the resident immune cells of the CNS, are indispensable for maintaining homeostasis through the secretion of neurotrophic factors and the clearance of cellular debris [9]. Under pathological conditions, however, sustained inflammatory stimuli induce a chronic, neurotoxic microglial phenotype characterized by excessive glutamate release and iron retention [10]. Toll-like receptor 2 (TLR2) plays a critical role in this cascade. Upon exposure to damage-associated molecular patterns (DAMPs), TLR2 heterodimerizes and initiates downstream inflammatory signaling via a myeloid differentiation primary response 88 (MyD88)-dependent pathway [[11], [12], [13]]. Accumulating evidence directly implicates TLR2 in NDDs pathogenesis. TLR2 expression is significantly upregulated across multiple brain regions in PD patients and correlates with α-synuclein accumulation [14]. Furthermore, tau protein has been shown to drive microglial activation and subsequent inflammation via TLR2 signaling; notably, blocking the TLR2-MyD88 interaction effectively counteracts tau-induced pathology in PS19 mice [15]. Although TLR2-targeted therapeutics are actively being pursued, none have yet achieved definitive clinical efficacy. Tomaralimab, a humanized anti-TLR2 monoclonal antibody, demonstrated acceptable tolerability and successfully blocked downstream inflammatory responses in a Phase I trial for kidney transplant rejection [16,17]. Recent Phase I studies investigating Tomaralimab for PD (NCT05790382) and multiple system atrophy (NCT06934941) were initiated in 2023 and 2024, respectively, but their final results remain pending. Therefore, identifying novel, small-molecule agents capable of effectively mitigating TLR2-mediated neuroinflammation is an urgent priority.

10,11-Dehydrocurvularin (DCV) is a naturally occurring benzanolide compound consisting of a resorcinol ring fused to a 12-membered macrolide (Fig. 1A), produced as a secondary metabolite by various fungal species. While DCV possesses a broad spectrum of recognized biological activities, including anticancer, antimicrobial, anti-inflammatory, and antioxidant properties [18]. However, its potential capacity in mitigating neuroinflammation and its precise underlying mechanisms remain unexplored. In this study, we investigated the anti-neuroinflammatory properties of DCV in lipopolysaccharide (LPS)-stimulated BV2 microglial cells and elucidated its dependence on the TLR2 signaling.

**Fig. 1:**
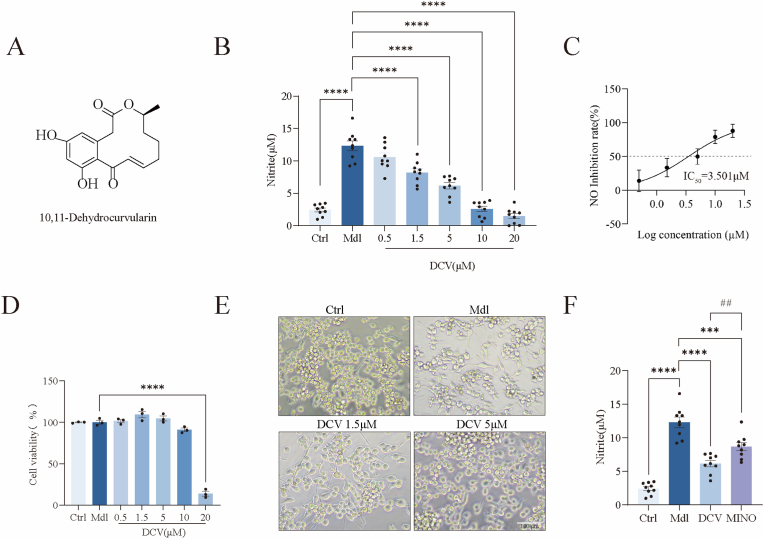
DCV inhibits NO production and ameliorates LPS-induced morphological changes in BV2 cells. (A) Chemical structure of DCV. (B) NO and nitrite levels in BV2 cell culture supernatants measured by Griess assay, n = 9. (C) NO inhibition rate and half-maximal inhibitory concentration (IC_50_) of DCV, n = 9. (D) Effect of DCV on BV2 cell viability assessed by CCK-8 assay, n = 3. (E) Representative bright-field images of BV2 cells across different treatment groups (scale bar = 100 μm). (F) Comparative anti-inflammatory efficacy of DCV and the positive control MINO, n = 9. “Mdl” denotes the model group treated with LPS alone. Data are presented as mean ± SEM. Comparisons between two groups were performed using Student's t-test. Multiple comparisons among groups were performed using one-way analysis of variance (ANOVA) followed by Dunnett's test. *∗∗∗P* < 0.001, *∗∗∗∗P* < 0.0001 vs. the Mdl group; ^*##*^*P* < 0.01 vs. the DCV group.

## 2 Materials and methods

### 2.1 Cell culture and compounds treatment

Murine microglial BV2 cells were obtained from the Cell Culture Center of the Institute of Basic Medical Sciences, Chinese Academy of Medical Sciences (Beijing, China), and murine microglial N9 cells were purchased from Wuhan Enki Life Technology Co., Ltd. (Enki, CXM00283). Both cell lines were maintained in a humidified atmosphere (5% CO_2_, 37°C) using high-glucose DMEM (Basalmedia, L110KJ). The culture medium was supplemented with 10% fetal bovine serum (FBS; Gibco, A5669701) and 1% penicillin-streptomycin solution (MeilunBio, MA0110). Cells were seeded into multi-well plates and allowed to adhere for 24 h prior to experimental treatments. The control group received vehicle throughout the experiment. For experimental groups, cells were pretreated with various concentrations of DCV, minocycline (MINO; MCE, HY-17412), or an equivalent volume of vehicle for 2 h before stimulation with 1 μg/mL lipopolysaccharide (LPS; MCE, HY-D1056). The experimental condition treated with LPS alone is referred to as the Model (Mdl) group. To investigate the functional involvement of TLR2, the TLR2 agonist Pam3CSK4 (Selleck, E7472) was co-administered alongside LPS at a final concentration of 5 μg/mL in the designated groups. For pharmacological inhibition experiments, cells were pretreated with 100 μg/mL of the TLR2 inhibitor C29 (MCE, HY-100461) or its vehicle for 2 h prior to stimulation with 1 μg/mL LPS alone or in combination with DCV. All downstream assays were performed after 36 h of LPS stimulation.

### 2.2 Nitric oxide (NO) assay

Nitrite accumulation in the culture supernatant was quantified as an indicator of NO production. Following the experimental treatments, culture supernatants were collected, and nitrite levels were quantified using Griess reagent kit (Beyotime, S0021 M) according to the manufacturer's instructions. Absorbance was measured at 548 nm using a microplate reader (Molecular Devices, SpectraMax Plus 384). Nitric oxide concentrations were extrapolated using a sodium nitrite standard curve.

### 2.3 Cell viability assay

To evaluate potential cytotoxicity, BV2 cells were pretreated with varying concentrations of DCV for 2 h followed by 36 h of LPS stimulation. The culture medium was then replaced with fresh complete medium containing 10% CCK-8 reagent (Life-ilab, AC11L054), and the cells were incubated at 37°C for 1 h. Absorbance was recorded at 450 nm using a microplate reader. Cell viability was calculated using the following formula: Cell viability (%) = (OD value of experimental group − OD value of blank group)/(OD value of control group − OD value of blank group) × 100%.

### 2.4 siRNA-mediated knock down of TLR2

To optimize knockdown efficiency, BV2 cells were transfected with three distinct candidate TLR2 small interfering RNAs (siRNA-1, siRNA-2, and siRNA-3) at a concentration of 60 nM using Lipofectamine RNAiMAX reagent. At 6 h post-transfection, the medium was replaced with fresh complete medium, and the cells were stimulated with LPS (1 μg/mL) for 36 h before total protein isolation. Knockdown efficiency was evaluated via Western blotting, and siRNA-1 was selected for subsequent functional validation. For the definitive functional assays, BV2 cells were transfected with 60 nM TLR2 siRNA-1. At 6 h post-transfection, the medium was replaced with fresh complete medium containing 5 μM DCV, and the cells were incubated for 2 h. Cells were then stimulated with 1 μg/mL LPS for 36 h, after which the culture supernatant was collected for NO quantification. The specific siRNA sequences utilized were:

TLR2 siRNA-1: (Sense) 5′-GGAAAUGUAGAGACUUUCAGUdTdT-3'; (Antisense) 5′-UGAAAGUCUCUACAUUUCCUAdTdT-3'

TLR2 siRNA-2: (Sense) 5′-GCAGUAAGUACUUUCUCUAAAdTdT-3'; (Antisense) 5′-UAGAGAAAGUACUUACUGCAUdTdT-3'

TLR2 siRNA-3: (Sense) 5′-CAGUAAGUACUUUCUCUAAAGdTdT-3'; (Antisense) 5′-UUAGAGAAAGUACUUACUGCAdTdT-3'

### 2.5 Western blotting

Cells were harvested in ice-cold PBS and lysed using RIPA buffer (Epizyme Biotech, PC101) supplemented with protease and phosphatase inhibitor cocktails. The lysates were cleared by centrifugation at 15,000× g for 15 min at 4°C, and protein concentrations were normalized. Samples were denatured at 100°C for 5-10 min and loaded onto 10% SDS-PAGE. Separated proteins were transferred to nitrocellulose membranes, blocked with 5% non-fat milk in TBST for 2 h at room temperature, and incubated overnight at 4°C with primary antibodies against NLRP3 (CST, #15101), TLR2 (Abcam, ab209217), MyD88 (CST, #4283S), TLR4 (Starter, S0B6179), apoptosis-associated speck-like protein containing a CARD (ASC, Starter, S0B1370), caspase-1 (CST, #89332S), cleaved caspase-1 (CST, #24232S), IL-1β (CST, #12242S), Gasdermin D (CST, #39754S), GAPDH (CST, #5174S) and β-actin (Starter, S0B0005). All primary antibodies were used at a dilution of 1:2000, except for β-actin and GAPDH (1:5000). Following three washes with TBST, membranes were incubated with HRP-conjugated secondary antibodies (1:5000, ABclonal, AS014) for 2 h at room temperature. Protein bands were visualized using YLESA enhanced chemiluminescence (ECL) reagents and quantified via optical density analysis using eBlot 14 software.

### 2.6 RT-qPCR

To quantify gene expression, total RNA was purified using the SteadyPure Quick RNA Extraction Kit (Accurate Biology, AG21023) and reverse-transcribed into cDNA with the Evo M-MLV RT Kit (Accurate Biology, AG11705). Amplification reactions were set up with SYBR Green Premix Pro Taq HS qPCR Kit (Accurate Biology, AG11701) under the following thermocycling conditions: initial denaturation at 95°C for 30 s, followed by 40 cycles of 95°C for 5 s and 60°C for 30 s. The specific primer sequences (Forward/Reverse, 5′-3′) used in this study targeted: IL-1β (TCGCAGCAGCACATCAACAAG/TCCACGGGAAAGACACAGGTAG), IL-6 (CCTTCTTGGGACTGATGCTGGTG/TGGTATCCTCTGTGAAGTCTCCTCTC), iNOS (TTGATGTGCTGCCTCTGGTCTTG/GCTCCTGGAACCACTCGTACTTG), COX2 (GAACCTGCAGTTTGCTGTGG/ACTCTGTTGTGCTCCCGAAG), TNF-α (ATGGCCTCCCTCTCATCAGT/AAGGTACAACCCATCGGCTG), Ccl2 (TGCCCTAAGGTCTTCAGCAC/ACTGTCACACTGGTCACTCC), Cxcl10 (TGCCGTCATTTTCTGCCTCA/CAAGCTTCCCTATGGCCCTC), CD86 (GCAAGGTCACCCGAAACCTA/TGTCAGCGTTACTATCCCGC), CD206 (AAAAGGCATGCGTTGCACAT/ACCTTGCCTGGGACAAACAA), Arg-1 (CTGGGGATTGGCAAGGTGAT/CTGTGATGCCCCAGATGGTT), Ccl12 (TATTGGCTGGACCAGATGCG/CCGGACGTGAATCTTCTGCT), Rsad2 (TTCTGAACTGTACCGGTGGC/CGCCACGCTTCAGAAACATC), Il36g (TCAGCGTGACTATCCTCCCA/AGCAGCAAAGTAGGGTGTCC), Ppp1r15a (CTCTGCCTTGTGGAAGCTGA/CCTGTGTGCCTCTACCTTGG) and GAPDH (AAGAAGGTGGTGAAGCAGGCATC/CGGCATCGAAGGTGGAAGAGTG).

### 2.7 Enzyme-linked immunosorbent assay (ELISA)

Murine IL-1β and IL-6 levels were measured via DuoSet ELISA kits (R&D Systems, DY401-05/DY406-05) according to the manufacturer's instructions. Briefly, ELISA plates were coated overnight with capture antibodies at room temperature, followed by blocking for 1 h. Samples and standards were then loaded and incubated for 2 h. Next, the plates were sequentially incubated with biotinylated detection antibodies for 2 h and streptavidin-HRP for 20 min. Between each incubation step, the plates were washed three times with PBS. After adding the substrate solution and incubating in the dark for 20 min, the enzymatic reaction was terminated with stop solution. Absorbance was immediately measured at 450 nm. Target protein concentrations were calculated using standard curves generated from serial dilutions.

### 2.8 Transcriptome sequencing and data analysis

Total RNA was extracted from cell pellets using the Adazol Total RNA Extraction Reagent (Adamas-life, G8011) strictly following the manufacturer's protocol. The extracted RNA was preserved on dry ice and shipped to Shanghai Majorbio Bio-Pharm Technology Co., Ltd. for library preparation and high-throughput RNA sequencing.

Bioinformatic processing was conducted via the nfcore-rnaseq pipeline (v3.14.0). Initial quality control of raw reads was performed with FastQC, after which TrimGalore was employed to remove adapters and filter out low-quality sequences. Clean reads were mapped to the *Mus musculus* reference genome (GRCm39) using the STAR aligner. Subsequently, gene-level counts were quantified using Salmon based on a pseudo-alignment approach. For downstream analysis, the nfcore-differentia labundance pipeline (v1.5.0) was utilized. Differential expression profiling was executed in DESeq2, incorporating transcript length data into the count matrix. Genes were retained for analysis if they met a minimum abundance of 20 counts and were present in at least 50% of the samples. Statistical significance was determined using the Benjamini–Hochberg correction for multiple testing. Genes were classified as differentially expressed (DEGs) when they satisfied the criteria of an adjusted *P*-value (padj) < 0.05 and an absolute log2 fold change (|log_2_FC|) ≥ 1 (corresponding to a linear fold change ≥2). Finally, functional enrichment analyses covering Gene Ontology (GO), KEGG, Reactome, and WikiPathways were carried out using the gProfiler2 package. This step employed hypergeometric testing with false discovery rate (FDR) correction via the Benjamini–Hochberg method.

### 2.9 Statistical analysis

All experiments were performed independently at least three times to ensure reproducibility. Statistical analysis and data visualization were conducted using GraphPad Prism software (version 9.5.1). Differences between two experimental conditions were evaluated with the Student's t-test, whereas multi-group comparisons were analyzed via one-way analysis of variance (ANOVA) followed by Dunnett's multiple comparisons post-hoc test. All data are shown as mean ± standard error of the mean (SEM), with statistical significance was defined as *P* < 0.05.

## 3 Results

### 3.1 DCV inhibits the inflammatory phenotype of LPS-induced BV2 cells

An acute inflammation model was established *in vitro* by stimulating BV2 cells with LPS for 36 h. Previous studies have shown that microglia release substantial amounts of NO, which serves as a phenotypic marker of inflammation, upon induction by pro-inflammatory factors like LPS [19]. Compared with the control group, NO production in the LPS-induced model group increased from 2.45 ± 0.89 μM to 12.31 ± 2.32 μM (*P* < 0.0001). Compared with the model group, treatment with 0.5, 1.5, 5, 10 and 20 μM of DCV in LPS-stimulated cells reduced NO concentrations to 10.6 ± 1.96 μM, 8.2 ± 1.68 μM, 6.17 ± 1.41 μM, 2.59 ± 1.22 μM, and 1.49 ± 1.2 μM, respectively (*P* = 0.1185, *P* < 0.0001, *P* < 0.0001, *P* < 0.0001, *P* < 0.0001, respectively) (Fig. 1B). The half-maximal inhibitory concentration (IC_50_) was calculated to be 3.501 μM (Fig. 1C). To exclude cytotoxicity, we assessed BV2 cell viability using a CCK-8 assay. DCV at concentrations ≤10 μM exhibited no toxicity under LPS stimulation, whereas 20 μM significantly reduced viability (Fig. 1D). Accordingly, subsequent experiments utilized the non-toxic concentration range of 0.5-5 μM to ensure that the observed anti-inflammatory effects were not due to cell death.

Bright-field microscopy at 20× magnification revealed that DCV effectively mitigated the LPS-induced morphological changes in BV2 cells, including soma swelling and abnormal process extension (Fig. 1E). Notably, at 5 μM, DCV reduced NO levels from 12.31 ± 2.32 μM (Mdl group) to 6.17 ± 1.41 μM (*P* < 0.0001), which outperformed the positive control minocycline (MINO), which reduced NO levels to 8.7 ± 1.8 μM (*P* = 0.0002). Therefore, DCV demonstrated a significantly greater inhibitory effect on NO production compared to MINO (*P* = 0.0043) (Fig. 1F).

### 3.2 DCV inhibits the production of inflammatory cytokines and promotes cellular repair function

Cytokines of the interleukin (IL) family, particularly IL-1β and IL-6, serve as primary drivers of pathological neuroinflammation [20]. To explore the underlying protective mechanisms of DCV, we measured the expression profiles of key inflammatory markers following LPS stimulation and DCV intervention. Compared with the control group, IL-1β protein concentration in the LPS-induced BV2 cells increased from 233.91 ± 76.48 pg/mL to 8987.13 ± 702 pg/mL (*P* < 0.0001). Compared with the model group, treatment with 0.5, 1.5 and 5 μM DCV reduced IL-1β protein concentrations to 8273.04 ± 1259.11 pg/mL, 7239.29 ± 1834.86 pg/mL and 5999.96 ± 1059.95 pg/mL, respectively (*P* = 0.7916, *P* = 0.1425 and *P* = 0.0076, respectively) (Fig. 2A). Similarly, IL-6 secretion surged from undetectable levels (0 pg/mL) in the control group to 2690.16 ± 234.88 pg/mL in the model group (*P* < 0.0001), whereas 0.5, 1.5 and 5 μM DCV reduced IL-6 protein levels to 1940.49 ± 316.24 pg/mL, 934.44 ± 81.79 pg/mL and 165.69 ± 53.38 pg/mL, respectively (*P* = 0.0001, *P* < 0.0001 and *P* < 0.0001, respectively) (Fig. 2B). At the transcriptional level, relative IL-1β expression in the model group increased from 1.01 ± 0.13 to 116.91 ± 18.94 relative to controls (*P* < 0.0001). Treatment with 0.5, 1.5 and 5 μM DCV reduced IL-1β mRNA levels to 93.62 ± 24.54, 95.76 ± 21.91 and 60.86 ± 12.9, respectively (*P* = 0.2422, *P* = 0.3144 and *P* = 0.0018, respectively) (Fig. 2C). Relative IL-6 mRNA expression in the model group increased from 1.02 ± 0.25 to 566 ± 53.22 relative to controls (*P* < 0.0001), and treatment with 0.5, 1.5 and 5 μM DCV reduced it to 388.19 ± 97.07, 209.27 ± 21.95 and 98.81 ± 13.14, respectively (*P* = 0.0006, *P* < 0.0001 and *P* < 0.0001, respectively) (Fig. 2D). Furthermore, LPS also up-regulated the transcription of the inflammatory mediators iNOS, COX2, TNF-α, Ccl2 and Cxcl10, from 1.06 ± 0.11 to 6.019 ± 0.63, from 1.01 ± 0.42 to 4.81 ± 2.12, from 1.02 ± 0.25 to 15.75 ± 1.89, from 1.02 ± 0.23 to 35.98 ± 9.75 and from 1.02 ± 0.26 to 9.48 ± 3.08, respectively (*P* = 0.0011, *P* < 0.0001, *P* < 0.0001, *P* < 0.0001 and *P* = 0.0002, respectively). Conversely, DCV treatment markedly attenuated these LPS-induced elevations, reducing the expression to 3.13 ± 0.57, 3.00 ± 0.95, 10.94 ± 1.20, 16.45 ± 2.12 and 4.72 ± 0.56, respectively (*P* = 0.0269, *P* = 0.0011, *P* = 0.0010, *P* = 0.0018 and *P* = 0.0088) (Fig. 2E–I).

**Fig. 2:**
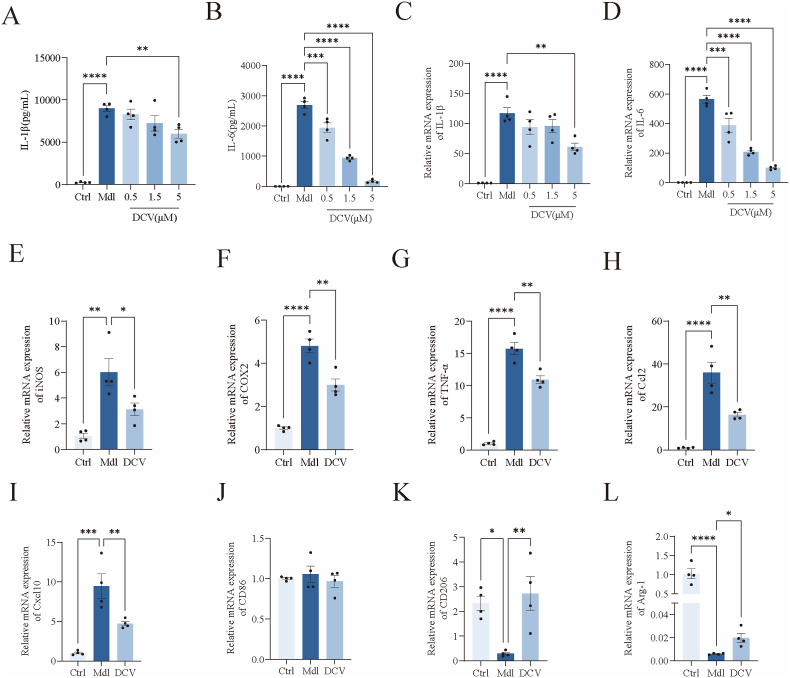
DCV attenuates pro-inflammatory cytokine secretion and promotes a reparative microglial phenotype. (A) IL-1β protein levels measured by ELISA, n = 4. (B) IL-6 protein levels measured by ELISA, n = 4. (C–L) Relative mRNA expression levels of IL-1β, IL-6, iNOS, COX2, TNF-α, Ccl2, Cxcl10, CD86, CD206, and Arg-1 quantified by RT-qPCR, n = 4. “Mdl” denotes the model group treated with LPS alone. “DCV” represents the group co-treated with LPS and 5 μM DCV. Data are presented as mean ± SEM. Multiple comparisons among groups were performed using one-way ANOVA followed by Dunnett's test. *∗P* < 0.05, *∗∗P* < 0.01, *∗∗∗P* < 0.001, *∗∗∗∗P* < 0.0001 vs. the Mdl group.

Intriguingly, neither LPS nor DCV significantly affected the transcriptional level of CD86 (Fig. 2J). However, LPS markedly downregulated the M2-type markers CD206 and Arg-1, from 2.33 ± 0.58 to 0.29 ± 0.36 (*P* = 0.0163) and from 1.024 ± 0.259 to 0.0059 ± 0.0007 (*P* < 0.0001), respectively. DCV treatment effectively reversed these LPS-induced suppressions, restoring CD206 expression to 2.72 ± 1.38 (*P* = 0.0062) and Arg-1 expression to 0.0200 ± 0.0078 (*P* = 0.0115) (Fig. 2K and L). Collectively, these findings suggest that DCV promotes a phenotypic shift of microglia from a pro-inflammatory state toward a reparative, neuroprotective phenotype.

### 3.3 DCV exerts anti-neuroinflammatory activity by inhibiting the TLR2/MyD88/NLRP3 pathway

**Fig. 3:**
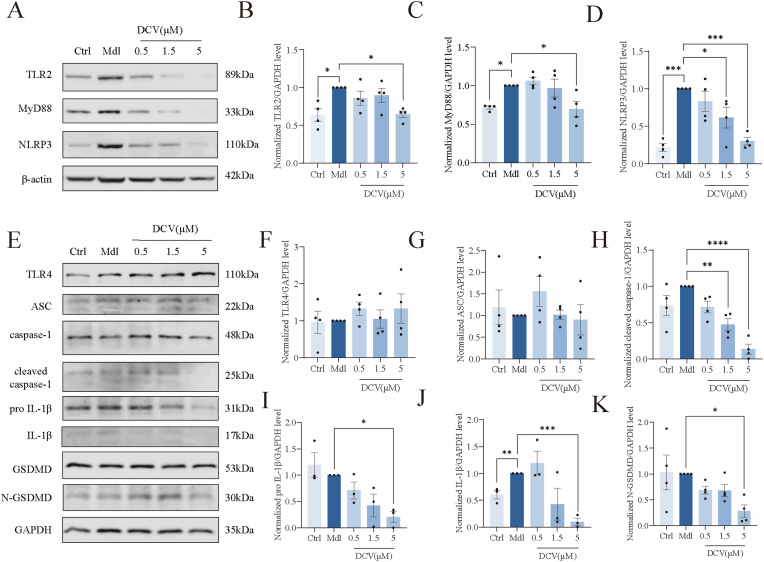
DCV suppresses the activation of the TLR2/MyD88/NLRP3 signaling cascade and NLRP3 inflammasome. (A) Representative Western blot images of TLR2, MyD88, NLRP3 and β-actin. Quantitative analysis of (B) TLR2/β-actin, n = 4, (C) MyD88/β-actin, n = 4 and (D) NLRP3/β-actin, n = 4. (E) Representative Western blot images of TLR4, ASC, caspase-1, cleaved caspase-1, pro-IL-1β, mature IL-1β, GSDMD, N-GSDMD and GAPDH. Quantitative analysis of (F) TLR4/GAPDH, n = 4, (G) ASC/GAPDH, n = 4, (H) cleaved caspase-1/GAPDH, n = 4, (I) pro IL-1β/GAPDH, n = 3, (J) IL-1β/GAPDH, n = 3 and (K) N-GSDMD/GAPDH, n = 4. “Mdl” denotes the model group treated with LPS alone. Data are presented as mean ± SEM. Multiple comparisons among groups were performed using one-way ANOVA followed by Dunnett's test. *∗P* < 0.05, *∗∗P* < 0.01, *∗∗∗P* < 0.001, *∗∗∗∗P* < 0.0001 vs. the Mdl group.

The TLR2/MyD88/NLRP3 signaling pathway is a vital cascade governing LPS-induced systemic and central inflammation [21]. To investigate whether DCV restrains neuroinflammation via this canonical axis, the protein levels of TLR2, MyD88, and NLRP3 were evaluated. LPS stimulation significantly upregulated the protein expression of TLR2, MyD88, and NLRP3 in BV2 cells (*P* = 0.0122, *P* = 0.0457 and *P* = 0.0001, respectively). Compared with the model group, treatment with 1.5 μM DCV significantly downregulated NLRP3 protein expression (*P* = 0.0343) while 5 μM DCV significantly downregulated all three core components: TLR2, MyD88, and NLRP3 (*P* = 0.0144, *P* = 0.0316 and *P* = 0.0003, respectively) (Fig. 3A–D). These results suggest that the anti-inflammatory action of DCV is mediated, at least in part, through the TLR2/MyD88/NLRP3 regulatory axis. We also examined whether DCV modulated TLR4 expression; in contrast to its effects on TLR2, DCV did not alter TLR4 protein levels (Fig. 3E and F). To further investigate whether DCV affects the functional activation of the NLRP3 inflammasome beyond downregulating overall NLRP3 protein levels, we assessed the proteolytic cleavage of caspase-1 and Gasdermin D (GSDMD), alongside the maturation of IL-1β. Western blot analysis revealed that treatment with DCV (5 μM) markedly attenuated the cleavage of caspase-1 (*P* < 0.0001) and GSDMD (*P* = 0.0323), and reduced mature IL-1β levels (*P* = 0.0002) (Fig. 3E–G–K). Although DCV did not significantly change the total protein level of the adapter ASC, the substantial inhibition of downstream executioners (cleaved caspase-1, N-GSDMD, and mature IL-1β) indicates that DCV effectively blocks the enzymatic activity and functional output of the NLRP3 inflammasome.

### 3.4 DCV exerts anti-inflammatory effects in a TLR2-dependent manner

**Fig. 4:**
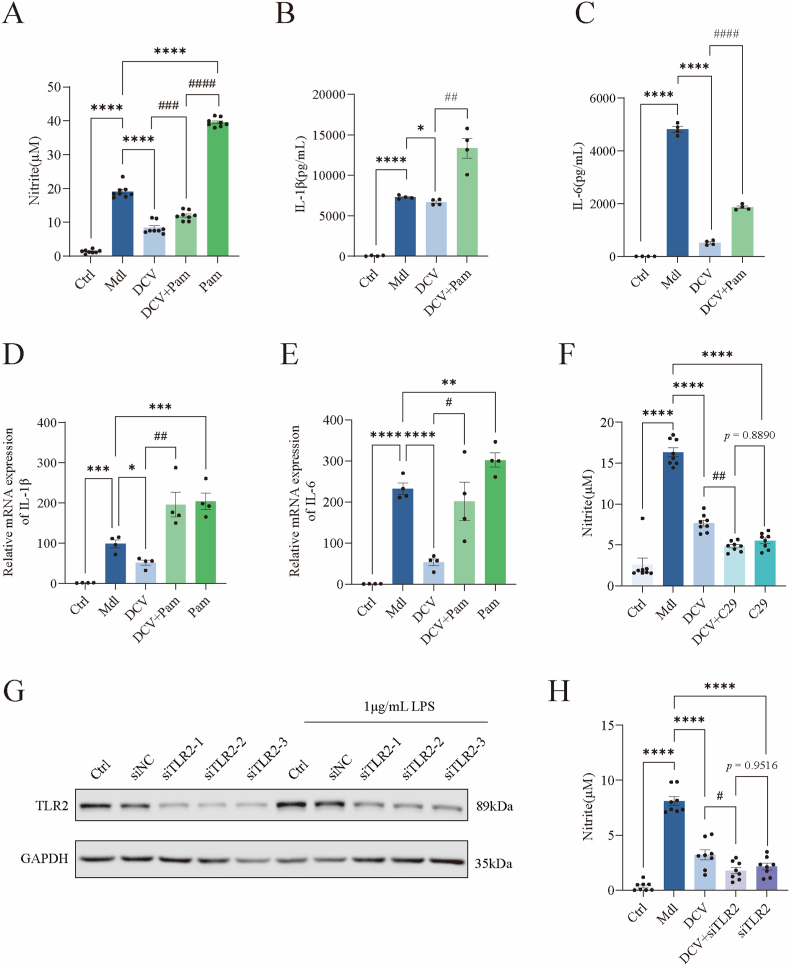
Intercepting or stimulating TLR2 confirms the TLR2-dependent mechanism of DCV. (A) NO/nitrite levels in BV2 supernatants following treatment with the TLR2 agonist Pam3CSK4 (Pam), n = 8. (B) IL-1β protein levels measured by ELISA, n = 4. (C) IL-6 protein levels measured by ELISA, n = 4. (D–E) Relative mRNA expression of IL-1β and IL-6, n = 4. (F) Supernatant NO/nitrite levels following treatment with the TLR2 inhibitor C29, n = 8. (G) Representative Western blot confirming successful siRNA-mediated knockdown of TLR2 (siTLR2). (H) Supernatant NO/nitrite levels in siTLR2-transfected BV2 cells, n = 8. “Mdl” denotes the model group treated with LPS alone. In panel H, the “Ctrl” and “Mdl” groups were transfected with negative control siRNA (siNC). “DCV” signifies groups treated with LPS +5 μM DCV. Data are presented as mean ± SEM. Comparisons between two groups were performed using Student's t-test. Multiple comparisons among groups were performed using one-way ANOVA followed by Dunnett's test. *∗P* < 0.05, *∗∗P* < 0.01, *∗∗∗P* < 0.001, *∗∗∗∗P* < 0.0001 vs. the Mdl group; ^*#*^*P* < 0.05, ^*##*^*P* < 0.01, ^*###*^*P* < 0.001, ^*####*^*P* < 0.0001 vs. the respective combination groups (DCV + Pam, DCV + C29, or DCV + siTLR2).

To confirm whether the anti-inflammatory properties of DCV depend strictly on TLR2 function, the specific TLR2 agonist Pam3CSK4 was co-administered. Compared with the DCV-treated group, the addition of Pam3CSK4 significantly elevated NO levels from 8.34 ± 1.84 μM to 12.01 ± 1.41 μM (*P* = 0.0001) (Fig. 4A). For IL-1β, Pam3CSK4 completely reversed the DCV-mediated suppression of protein secretion, raising it from 6685.93 ± 351.72 pg/mL to 13339.55 ± 2431.43 pg/mL (*P* = 0.0016) (Fig. 4B). Similarly, IL-6 protein suppression was reversed, increasing from 513.44 ± 89 pg/mL to 1858.99 ± 103.63 pg/mL (*P* < 0.0001) (Fig. 4C). At the mRNA level, Pam3CSK4 counteracted the inhibitory effect of DCV, restoring IL-1β transcripts from a relative expression of 51.02 ± 13.17 to 195.6 ± 62.21 (*P* = 0.0021) (Fig. 4D), and IL-6 transcripts from 53.51 ± 16.83 to 201.80 ± 93.44 (*P = *0.0107) (Fig. 4E). Additionally, Pam3CSK4 application reversed the DCV-mediated transcriptional downregulation of other key inflammatory markers, such as TNF-α, iNOS, and COX2 (Supplementary Fig. 1), suggesting that DCV likely exerts its anti-inflammatory effects through modulation of the TLR2 pathway. To strengthen the biological relevance, we validated the key findings in the N9 microglial cell line. We found that DCV significantly reduced NO production and downregulated IL-1β and IL-6 mRNA levels in N9 cells. Importantly, Pam3CSK4 reversed the anti-inflammatory effects of DCV in N9 cells (Supplementary Fig. 2). We next investigated whether the anti-inflammatory effect of DCV was affected by TLR2 inhibition. After 36 h of LPS induction, we found that C29 combined with DCV significantly reduced the level of NO compared to the model group (*P* = 0.0018), whereas C29 + DCV did not significantly alter NO levels compared with the C29 alone treatment group (*P* = 0.8890) (Fig. 4F). Similarly, we knocked down the expression of TLR2 protein in BV2 cells using siRNA, and then found that TLR2 knockdown combined with DCV significantly reduced NO levels compared to the Mdl group (*P* = 0.0244), whereas the TLR2 knockdown + DCV group did not significantly alter NO levels compared with the TLR2 knockdown alone group (*P* = 0.9516) (Fig. 4G and H). The above results support the conclusion that DCV exerts anti-inflammatory effects in a TLR2-dependent manner.

### 3.5 Transcriptomic profiling reveals DCV-mediated reprogramming of the inflammatory landscape

**Fig. 5:**
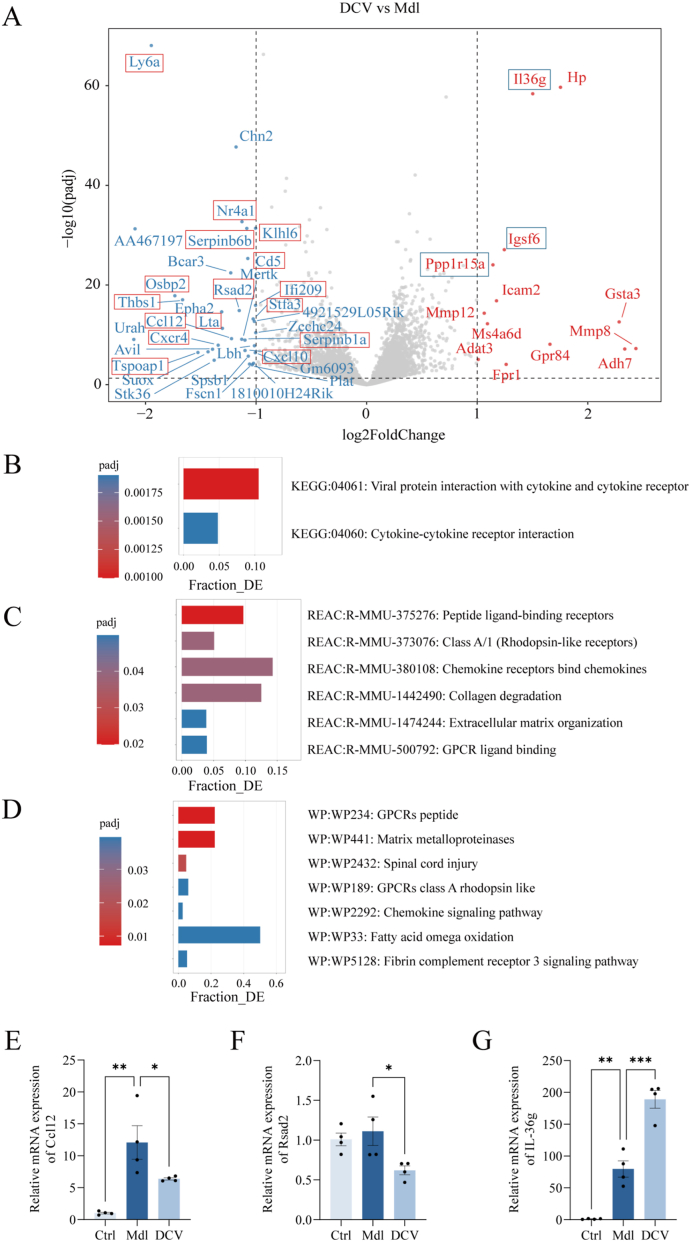
Transcriptomic analysis of gene expression changes in LPS-induced BV2 cells treated with DCV. (A) Volcano plot of differentially expressed genes. (B) KEGG pathway enrichment analysis. (C) REAC pathway enrichment analysis. (D) WP pathway enrichment analysis. (E–G) RT-qPCR validation of Ccl12, Rsad2, and Il36g mRNA levels, n = 4. “Mdl” denotes the model group treated with LPS alone. “DCV” represents the group treated with LPS +5 μM DCV. Data are presented as mean ± SEM. Comparisons between two groups were performed using Student's t-test. Multiple comparisons among groups were performed using one-way ANOVA followed by Dunnett's test. *∗P* < 0.05, *∗∗P* < 0.01, and *∗∗∗P* < 0.001 vs. the Mdl group.

Having established that DCV acts through the canonical TLR2/MyD88/NLRP3 axis, we sought to map global transcriptional alterations to determine whether its regulatory effects extend beyond this path. BV2 cells were pretreated with 5 μM DCV for 2 h prior to LPS stimulation for 36 h, followed by total RNA extraction and high-throughput transcriptomic sequencing. As illustrated in the volcano plot (Fig. 5A), differential expression analysis revealed a distinct cluster of differentially expressed genes (DEGs) primarily enriched in immune regulation and inflammatory networks. Notably, DCV treatment significantly downregulated a panel of pivotal pro-inflammatory genes, including Rsad2, Nr4a1, Lta, Cd5, Tspoap1, Cxcl10, Ccl12, Serpinb6b, Klhl6, Ifi209, Serpinb1a, Stfa3, and Ly6a. The suppression of these genes, which are critical drivers of the innate immune response and chemokine signaling [[22], [23], [24], [25], [26], [27], [28], [29], [30], [31], [32], [33], [34]], directly correlates with the observed reduction in inflammatory markers (NO, IL-1β, IL-6). Conversely, a distinct subset of genes, including Il36g, Ppp1r15a, and Igsf6, remained upregulated or showed specific regulation patterns [[35], [36], [37]], suggesting that DCV induces gene-specific modifications rather than a non-specific transcriptomic shut down.

Concurrently, pathway enrichment analyses using KEGG, Reactome (REAC), and WikiPathways (WP) (Fig. 5B–D) highlighted significant alterations in pathways governing immune cell communication and signaling. Top enriched terms included “Cytokine-cytokine receptor interaction,” “Chemokine receptors bind chemokines,” and various GPCR-related pathways (e.g., “GPCR ligand binding,” “Peptide ligand-binding receptors”). Although the KEGG term “Viral protein interaction with cytokine and cytokine receptor” was enriched, this reflects the broader disruption of the cytokine network interface rather than a direct viral mechanism. Additionally, pathways such as “Chemokine signaling” and “Fibrin complement receptor 3 signaling” were prominently modulated. These findings suggest that beyond suppressing individual canonical cascades, DCV may engage broader immunomodulatory mechanisms by remodeling the chemokine and GPCR signaling networks in LPS-stimulated BV2 microglia. To validate these RNA-seq findings, we analyzed Ccl12, Rsad2, and Il36g transcript levels via RT-qPCR. In agreement with the transcriptomic data, DCV significantly downregulated Ccl12 (*P* = 0.0485) and Rsad2 (*P* = 0.0292) while upregulating Il36g (*P* = 0.0001) (Fig. 5E–G). These findings indicate that DCV achieves its therapeutic efficacy by selectively curbing the LPS-primed pro-inflammatory transcriptional program, helping shift microglia into a resolved, protective state.

## 4 Discussion

The pivotal role of Toll-like receptor 2 (TLR2) in the pathogenesis of neurodegenerative diseases (NDDs) has been extensively documented. For instance, the opioid receptor antagonist naltrexone attenuates oxycodone-induced, high-mobility group box 1 (HMGB1)-mediated neuroimmune crosstalk between oligodendrocytes and microglia via blocking the receptor for advanced glycation end products (RAGE) and TLR2 cascades [38]. Similarly, probiotics such as *Bifidobacterium animalis* HN019 and *Lactobacillus acidophilus* NCFM exert neuroprotective effects in 1-Methyl-4-phenyl-1,2,3,6-tetrahydropyridine (MPTP)-induced Parkinson's disease models by inhibiting the TLR2/TLR4-NF-κB pathway activation within both the colon and substantia nigra [39]. Genetic ablation of TLR2 has been reported to reduce tau pathology and microglial activation in rTg4510 tau transgenic mice. Furthermore, therapeutic blockade of TLR2 using the monoclonal antibody Tomaralimab dose-dependently inhibits receptor activation and inflammatory responses, dramatically mitigating tau spread and memory deficits *in vivo* [40]. Advanced delivery systems, such as poly (lactic-co-glycolic acid) nanoparticles carrying an anti-TLR2 single-chain variable fragment (scFv33), successfully reduce hippocampal Aβ plaques and improving cognitive function in 5xFAD mice following cisterna magna administration [41].

Driven by these insights, the development of novel therapeutics targeting TLR2-mediated neuroinflammation is accelerating. Synthetic peptides like GSP1-111 alleviate neuroinflammation in LPS-stimulated BV2 cells and murine models by downregulating TLR2 expression, concurrently ameliorating depressive-like behaviors [42]. The histone deacetylase inhibitor givinostat improves pathological features of experimental autoimmune encephalomyelitis (EAE) by blocking the TLR2/MyD88/IRF5 axis, thereby reducing inflammation and glial activation in the spinal cord [43]. Additionally, natural formulations, such as the *Gastrodia elata* compound formula, have been found to suppress the TLR2/MyD88/NF-κB cascade, attenuating neuroinflammation and cognitive impairment induced by chronic sleep restriction [44]. The anti-neuroinflammatory properties of other agents, such as vortioxetine, Xixin decoction, asiatic acid, and the essential oil of *Acorus tatarinowii* rhizoma, are also increasingly attributed to TLR2 inhibition [[45], [46], [47], [48]]. Given this therapeutic landscape, it is necessary to continue developing new TLR2-dependent drugs for NDDs to meet the increasingly urgent clinical needs. This study demonstrates that 10,11-dehydrocurvularin (DCV) effectively attenuates lipopolysaccharide (LPS)-induced inflammatory responses in BV2 microglial cells by suppressing the TLR2/MyD88/NLRP3 signaling pathway. Our results identify DCV as a potent natural agent capable of mitigating neuroinflammation, a cornerstone pathology of NDDs.

To map the broader systemic impacts of DCV on the microglial activation landscape, we performed transcriptomic profiling. Treatment with DCV significantly reprogrammed the gene expression profile of LPS-stimulated BV2 cells. Functional annotation revealed that DCV selectively suppressed a core cohort of pro-inflammatory genes, including Rsad2, Nr4a1, Lta, Cd5, Tspoap1, Cxcl10, Ccl12, Serpinb6b, Klhl6, Ifi209, Serpinb1a, Stfa3, and Ly6a. These genes are critical drivers of innate immune responses and chemokine signaling; their downregulation correlates directly with the observed reduction in inflammatory markers (NO, IL-1β, IL-6). Conversely, a distinct subset of genes, including Il36g, Ppp1r15a, and Igsf6, exhibited unique regulation patterns, suggesting that DCV does not induce a blanket suppression of transcription but rather remodels the microglial phenotype from a hyper-inflammatory state to a resolved, protective state. Enrichment analyses corroborated these findings, highlighting significant alterations in pathways governing cytokine-cytokine receptor interactions and chemokine signaling. Collectively, these data suggest that DCV engages broad immunomodulatory mechanisms beyond canonical pathway inhibition, potentially offering a more nuanced therapeutic approach to resolving neuroinflammation.

Despite these encouraging insights, several limitations of this study should be noted. First, while our data confirm that DCV inhibits the TLR2/MyD88/NLRP3 axis, it remains unclear whether DCV directly binds to the TLR2 pocket or regulates its expression indirectly through upstream modulators. Second, due to the relatively low biosynthetic yield of DCV, this study was limited to *in vitro* models; consequently, the pharmacodynamic effects and therapeutic efficacy of DCV in complex *in vivo* disease environments remain to be established. Future research will focus on: (1) validating the necessity of TLR2 for DCV's effects using TLR2-knockout animal models (2) employing target fishing techniques (e.g., CETSA, DARTS); to identify the direct molecular target of DCV; (3) evaluating the efficacy of DCV in transgenic animal models of Alzheimer's and Parkinson's diseases; and (4) assessing the selectivity of DCV against other Toll-like receptors to ensure safety and specificity. While further *in vivo* studies are required to validate its pharmacokinetic properties and therapeutic efficacy, our results suggest that DCV represents a promising lead compound for the development of treatments for neuroinflammation-associated disorders.

## 5 Conclusion

DCV effectively attenuates LPS-induced neuroinflammation in microglial BV2 cells by inhibiting the TLR2/MyD88/NLRP3 signaling axis and downregulating the expression of some NLRP3 inflammasome components, as well as pro-inflammatory cytokines and chemokines. Notably, the reversal of its anti-inflammatory effects by the TLR2 agonist Pam3CSK4 critically suggests TLR2 as a key mediator of DCV activity. This TLR2-dependent mechanism was further confirmed by functional inhibition using the specific TLR2 inhibitor C29 and siRNA-mediated TLR2 knockdown. Furthermore, transcriptomic analysis revealed that DCV exerts broad immunomodulatory and anti-inflammatory effects on LPS-stimulated BV2 cells. Collectively, these findings highlight DCV as a promising candidate lead compound for neuroinflammatory conditions.

## CRediT authorship contribution statement

**Chenggang Tian:** Data curation, Formal analysis, Investigation, Methodology, Validation, Writing – original draft. **Jian Sun:** Investigation. **Jinting Wang:** Resources. **Yu Wang:** Data curation, Investigation. **Peifeng Wan:** Investigation. **Guangqiang Sun:** Investigation, Resources. **Yanling Mu:** Supervision. **Meiyu Geng:** Funding acquisition, Resources. **Baofu Xu:** Conceptualization, Resources. **Yu Zhang:** Conceptualization, Funding acquisition, Investigation, Project administration, Supervision, Writing – review & editing.

## Declaration of competing interest

The authors declare no competing interests.

## Figures and Tables

**figs1:**
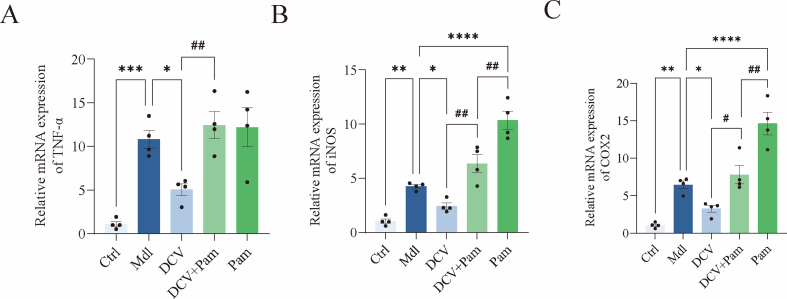


**figs2:**
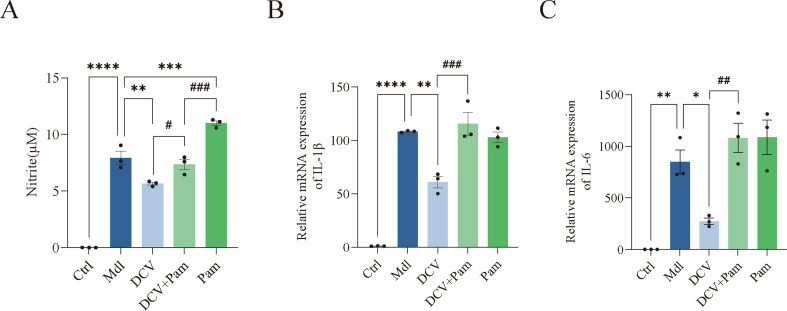


## Data Availability

Data will be made available on request.

## References

[1] Shi F.D., Yong V.W. (2025). Neuroinflammation across neurological diseases. Science.

[2] Chen X., Firulyova M., Manis M., Herz J., Smirnov I., Aladyeva E., Wang C., Bao X., Finn M.B., Hu H., Shchukina I., Kim M.W., Yuede C.M., Kipnis J., Artyomov M.N., Ulrich J.D., Holtzman D.M. (2023). Microglia-mediated T cell infiltration drives neurodegeneration in tauopathy. Nature.

[3] Kouli A., Camacho M., Allinson K., Williams-Gray C.H. (2020). Neuroinflammation and protein pathology in Parkinson's disease dementia. Acta Neuropathol. Commun..

[4] Shi K., Tian D.C., Li Z.G., Ducruet A.F., Lawton M.T., Shi F.D. (2019). Global brain inflammation in stroke. Lancet Neurol..

[5] Beuker C., Strecker J.K., Rawal R., Schmidt-Pogoda A., Ruck T., Wiendl H., Klotz L., Schäbitz W.R., Sommer C.J., Minnerup H., Meuth S.G., Minnerup J. (2021). Immune cell infiltration into the brain after ischemic stroke in humans compared to mice and rats: a systematic review and meta-analysis. Transl Stroke Res.

[6] Lu Y., Guan L., Wu J., Yang Q., Zhang M., Zhou D., Yang H., Pan Y., Wang L., Qiu B., Liu C., Wang Y., Yang Y., Zhou X., Qu H., Liao X., Liu L., Zhao X., Bath P.M., Johnston S.C., Amarenco P., Turc G., Shi F.D., Wang Y., Wang Y. (2026). Efficacy and safety of minocycline in patients with acute ischaemic stroke (EMPHASIS): a multicentre, double-blind, randomised controlled trial. Lancet.

[7] Evola V., Parmar M.S. (2026). Targeting neuroinflammation in neurodegenerative disorders: the emerging potential of semaglutide. Inflamm. Res..

[8] Cummings J.L., Atri A., Feldman H.H., Hansson O., Sano M., Knop F.K., Johannsen P., León T., Scheltens P. (2025). Evoke and evoke+: design of two large-scale, double-blind, placebo-controlled, phase 3 studies evaluating efficacy, safety, and tolerability of semaglutide in early-stage symptomatic Alzheimer's disease. Alzheimers Res. Ther..

[9] Isik S., Yeman Kiyak B., Akbayir R., Seyhali R., Arpaci T. (2023). Microglia mediated neuroinflammation in Parkinson's Disease. Cells.

[10] Yildirim-Balatan C., Fenyi A., Besnault P., Gomez L., Sepulveda-Diaz J.E., Michel P.P., Melki R., Hunot S. (2024). Parkinson's disease-derived α-synuclein assemblies combined with chronic-type inflammatory cues promote a neurotoxic microglial phenotype. J. Neuroinflammation.

[11] Qi X.H., Chen P., Wang Y.J., Zhou Z.P., Liu X.C., Fang H., Wang C.W., Liu J., Liu R.Y., Liu H.K., Zhang Z.X., Zhou J.N. (2024). Increased cysteinyl-tRNA synthetase drives neuroinflammation in Alzheimer's disease. Transl. Neurodegener..

[12] Yang X.P., Huang J.H., Ye F.L., Yv Q.Y., Chen S., Li W.W., Zhu M. (2024). Echinacoside exerts neuroprotection via suppressing microglial α-synuclein/TLR2/NF-κB/NLRP3 axis in parkinsonian models. Phytomedicine.

[13] Fu J., Wu H. (2023). Structural mechanisms of NLRP3 inflammasome assembly and activation. Annu. Rev. Immunol..

[14] Dzamko N., Gysbers A., Perera G., Bahar A., Shankar A., Gao J., Fu Y., Halliday G.M. (2017). Toll-like receptor 2 is increased in neurons in Parkinson's disease brain and may contribute to alpha-synuclein pathology. Acta Neuropathol..

[15] Dutta D., Jana M., Paidi R.K., Majumder M., Raha S., Dasarathy S., Pahan K. (2023). Tau fibrils induce glial inflammation and neuropathology via TLR2 in Alzheimer's disease-related mouse models. J. Clin. Investig..

[16] Arslan F., Houtgraaf J.H., Keogh B., Kazemi K., de Jong R., McCormack W.J., O'Neill L.A., McGuirk P., Timmers L., Smeets M.B., Akeroyd L., Reilly M., Pasterkamp G., de Kleijn D.P. (2012). Treatment with OPN-305, a humanized anti-toll-like receptor-2 antibody, reduces myocardial ischemia/reperfusion injury in pigs. Circ Cardiovasc Interv.

[17] Reilly M., Miller R.M., Thomson M.H., Patris V., Ryle P., McLoughlin L., Mutch P., Gilboy P., Miller C., Broekema M., Keogh B., McCormack W., van de Wetering de Rooij J. (2013). Randomized, double-blind, placebo-controlled, dose-escalating phase I, healthy subjects study of intravenous OPN-305, a humanized anti-TLR2 antibody. Clin. Pharmacol. Ther..

[18] Zhou F., Zhou Y., Guo Z., Yu X., Deng Z. (2022). Review of 10,11-Dehydrocurvularin: synthesis, structural diversity, bioactivities and mechanisms. Mini Rev. Med. Chem..

[19] Ni C., Wang L., Bai Y., Huang F., Shi H., Wu H., Wu X., Huang J. (2025). Taurochenodeoxycholic acid activates autophagy and suppresses inflammatory responses in microglia of MPTP-induced Parkinson's disease mice via AMPK/mTOR, AKT/NFκB and Pink1/Parkin signaling pathways mediated by Takeda G protein-coupled receptor 5. Free Radic. Biol. Med..

[20] Khan A.W., Farooq M., Hwang M.J., Haseeb M., Choi S. (2023). Autoimmune neuroinflammatory diseases: role of interleukins. Int. J. Mol. Sci..

[21] Cui K., Wang J., Chen K., Ge W., Zhou J., Zhang R., Fan X. (2026). Astaxanthin protects against acute lung injury via dual modulation of Ca(2+)/CaMKIIα/NLRP3 and TLR2/MyD88/NLRP3 pathways. Int. Immunopharmacol..

[22] Wang Q., Wang S., Cui L., Zhang Y., Waterhouse G.I.N., Sun-Waterhouse D., Ma C., Kang W. (2025). Flammulina velutipes polysaccharide exerts immunomodulatory function involving RSAD2 to regulate the NF-κB/MAPK signaling pathway in RAW264.7 macrophage cells and in mouse spleen cells. Int. J. Biol. Macromol..

[23] Tao Y., Tang C., Wei J., Shan Y., Fang X., Li Y. (2023). Nr4a1 promotes renal interstitial fibrosis by regulating the p38 MAPK phosphorylation. Mol Med.

[24] Gutiérrez-Venegas G., Fernández-Rojas B., Rosas-Martínez M., Sánchez-Carballido M.A. (2020). Rutin prevents LTA induced oxidative changes in H9c2 cells. Prev Nutr Food Sci.

[25] Wada T. (2018). Downregulation of CD5 and dysregulated CD8(+) T-cell activation. Pediatr. Int..

[26] Galiègue S., Jbilo O., Combes T., Bribes E., Carayon P., Le Fur G., Casellas P. (1999). Cloning and characterization of PRAX-1. A new protein that specifically interacts with the peripheral benzodiazepine receptor. J. Biol. Chem..

[27] Bufi A.A., Di Stefano J., Papait A., Silini A.R., Parolini O., Ponsaerts P. (2025). The central role of CXCL10-CXCR3 signaling in neuroinflammation and neuropathology. Cytokine Growth Factor Rev..

[28] Lin B., Zhang H., Zhu P., Chen J., Li D., Zhou J., Zhang T., Chen Q., Tang C., Song X., Zeng H., Wang J., Zhang J., You Z., Ma X., Yu C. (2025). Bone marrow-derived CD169(+) macrophages promote autoimmune hepatitis by recruiting CCR2(+) monocytes via secreting CCL12. Exp. Mol. Med..

[29] Garzón-Tituaña M., Sierra-Monzón J.L., Comas L., Santiago L., Khaliulina-Ushakova T., Uranga-Murillo I., Ramirez-Labrada A., Tapia E., Morte-Romea E., Algarate S., Couty L., Camerer E., Bird P.I., Seral C., Luque P., Paño-Pardo J.R., Galvez E.M., Pardo J., Arias M. (2021). Granzyme A inhibition reduces inflammation and increases survival during abdominal sepsis. Theranostics.

[30] Pu Y., Cao X. (2026). KLHL6: a proteostatic guardian against T-cell exhaustion. Trends Immunol..

[31] Wang S., Bai J. (2022). Functions and roles of IFIX, a member of the human HIN-200 family, in human diseases. Mol. Cell. Biochem..

[32] Lan C., Fang G., Li X., Chen X., Chen Y., Hu T., Wang X., Cai H., Hao J., Li H., Zhang Y., Peng K., Xu Z., Yang D., Kang X., Xin Q., Yang Y. (2025). SerpinB1 targeting safeguards against pathological cardiac hypertrophy and remodelling by suppressing cardiomyocyte pyroptosis and inflammation initiation. Cardiovasc. Res..

[33] Lei L., Zhang X., Yang R., Jing H., Yuan Y., Chen Z., Gou Q., Zhao Z., Zhang J., Wang X. (2022). Host immune response to clinical hypervirulent Klebsiella pneumoniae pulmonary infections via transcriptome analysis. J. Immunol. Res..

[34] Shmerling M., Chalik M., Smorodinsky N.I., Meeker A., Roy S., Sagi-Assif O., Meshel T., Danilevsky A., Shomron N., Levinger S., Nishry B., Baruchi D., Shargorodsky A., Ziv R., Sarusi-Portuguez A., Lahav M., Ehrlich M., Braschi B., Bruford E., Witz I.P., Wreschner D.H. (2022). LY6S, a new IFN-Inducible human member of the Ly6a subfamily expressed by spleen cells and associated with inflammation and viral resistance. ImmunoHorizons.

[35] Meng X., Zhong Y., Kuang X., Zhang Y., Yang L., Cai Y., Wang F., He F., Xie H., Wang B., Li J. (2025). Targeting the STAT3/IL-36G signaling pathway can be a promising approach to treat rosacea. J. Adv. Res..

[36] Liu L., Li J., Yin P., Kong S., Li J. (2025). PPP1R15A promotes apoptosis, autophagy, and inflammatory response to exacerbate ischemic stroke through activation of TLR4/NF-κB pathway. J. Neurosci. Res..

[37] Wu Y., Zhang P., Shi T., Cao D., Pan W. (2024). Deficiency of immunoglobulin IgSF6 enhances antibacterial effects by promoting endoplasmic reticulum stress and the inflammatory response in intestinal macrophages. Mucosal Immunol..

[38] Maulik M., Rose C., Mallick S., Jones A., Dodge A., Chakraborty S., Carter A., Jeffers A., Ford M., Zhang E., Varnell B., Davis R., Mitra S. (2026). Oxycodone induces HMGB1-mediated neuroimmune crosstalk between oligodendrocytes and microglia. Neuroscience.

[39] Mo C., Qian Y., Zhang Y., He X., Lai Y., Xu S., Ai P., Yang X., Xiao Q. (2026). Neuroprotective effects of Bifidobacterium animalis HN019 and Lactobacillus acidophilus NCFM in MPTP-induced Parkinson's disease mice. Brain Behav. Immun..

[40] Kim Y., Ryu S.H., Hyun J., Cho Y.S., Jung Y.K. (2024). TLR2 immunotherapy suppresses neuroinflammation, tau spread, and memory loss in rTg4510 mice. Brain Behav. Immun..

[41] Lee S., Lee J., Jeon J., Lee H., Choi B., Hong J., Lee S.J. (2026). PLGA nanoparticle-based Anti-TLR2 scFv gene delivery for the treatment of Alzheimer's Disease. Exp. Neurobiol..

[42] Kim R.E., Mabunga D.F., Boo K.J., Kim D.H., Han S.H., Shin C.Y., Kwon K.J. (2024). GSP1-111 modulates the microglial M1/M2 phenotype by inhibition of toll-like receptor 2: a potential therapeutic strategy for depression. Int. J. Mol. Sci..

[43] Shen Y., Zhao J., Yang R., Yang H., Guo M., Ji B., Du G., Li L. (2024). Panobinostat Attenuates experimental autoimmune encephalomyelitis in mice via suppressing oxidative stress-related neuroinflammation and mitochondrial dysfunction. Int. J. Mol. Sci..

[44] Zhang Y., Chen F., Li X., Xu Y., Liu X., Barkat M.Q., Choudhary M.I., Chang Q., Jiang N., elata Gastrodia, sibiricum Polygonatum (2025). Poria cocos as a functional food formula: cognitive enhancement via modulation of hippocampal neuroinflammation and neuroprotection in sleep-restricted mice. Foods.

[45] Samur D.N., Yıldırım S., Maytalman E., Kalay M., Tanrıöver G., Özbey G. (2025). Vortioxetine attenuates rotenone-induced enteric neuroinflammation via modulation of the TLR2/S100B/RAGE signaling pathway in a rat model of Parkinson's disease. Neuropharmacology.

[46] Yang C., Zhao E., Zhang H., Duan L., Han X., Ding H., Cheng Y., Wang D., Lei X., Diwu Y. (2024). Xixin Decoction's novel mechanism for alleviating Alzheimer's disease cognitive dysfunction by modulating amyloid-β transport across the blood-brain barrier to reduce neuroinflammation. Front. Pharmacol..

[47] Zou W., Li J. (2026). Asiatic acid attenuates Salmonella typhimurium-Induced neuroinflammation and neuronal damage by inhibiting the TLR2/Notch and NF-κB pathway in Microglia. Int. J. Mol. Sci..

[48] Hu Y., Zhao Y., Mao Z., Yang J., Huang B., Miao J., Miao M. (2025). Inhalation of Acori Tatarinowii Rhizoma essential oil alleviates dyskinesia in Parkinson's disease rats through the regulation of neuroinflammation. J. Ethnopharmacol..

