# Supplementary material for: 10,11-Dehydrocurvularin attenuates LPS-induced neuroinflammation in BV2 cells by inhibiting the TLR2/MyD88/NLRP3 signaling pathway
Source: Biochem Biophys Rep. 2026 Jul 18;47:102718. doi: 10.1016/j.bbrep.2026.102718 (PMC13393164; doi:10.1016/j.bbrep.2026.102718)
Supplement: Multimedia component 1 [file mmc1.pdf]

Original western blot for four repeats in Fig 3 (A-D).

Fig. 3A

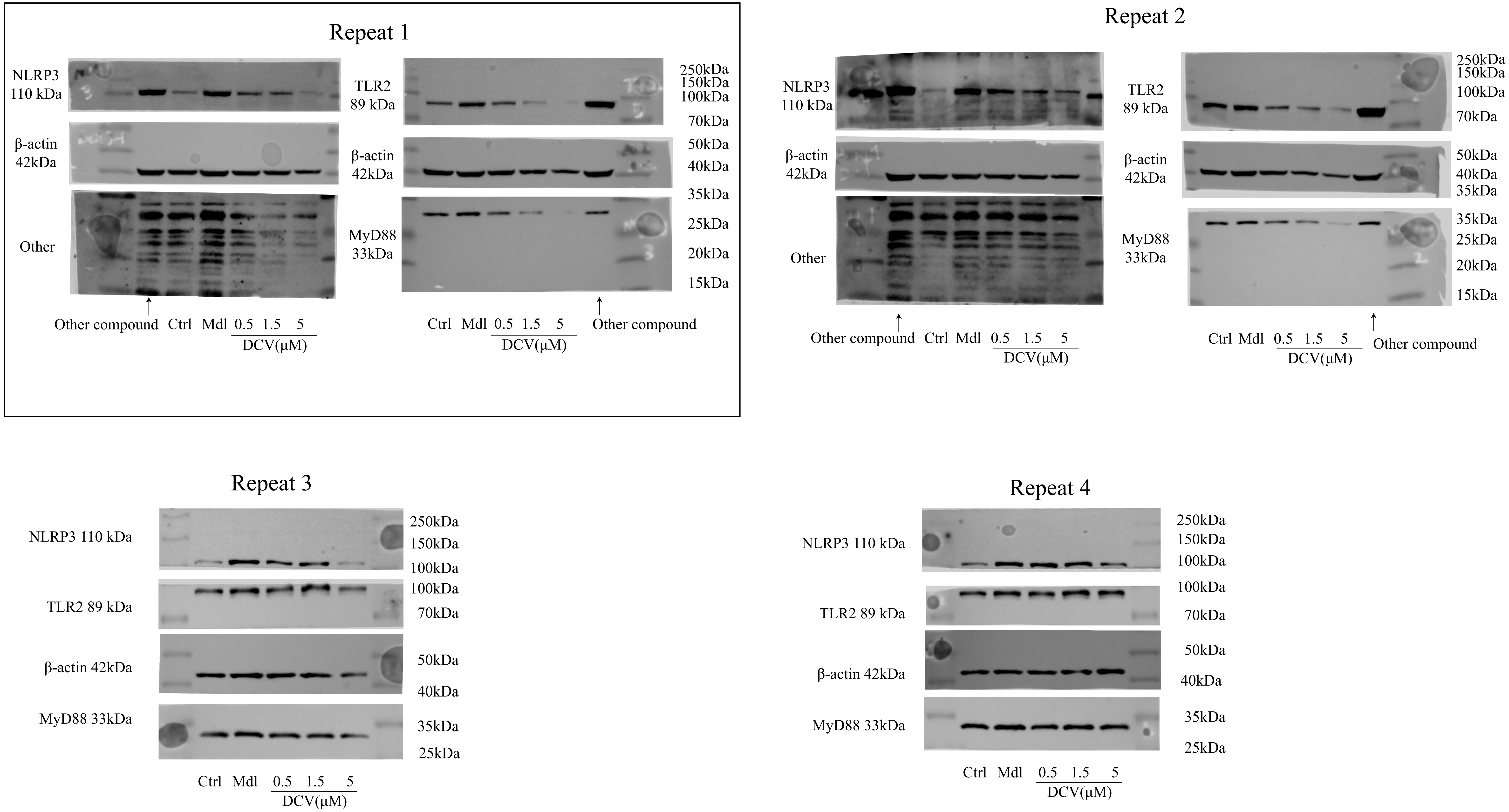

Original western blot for four repeats in Fig 3 (E, F, G, K)

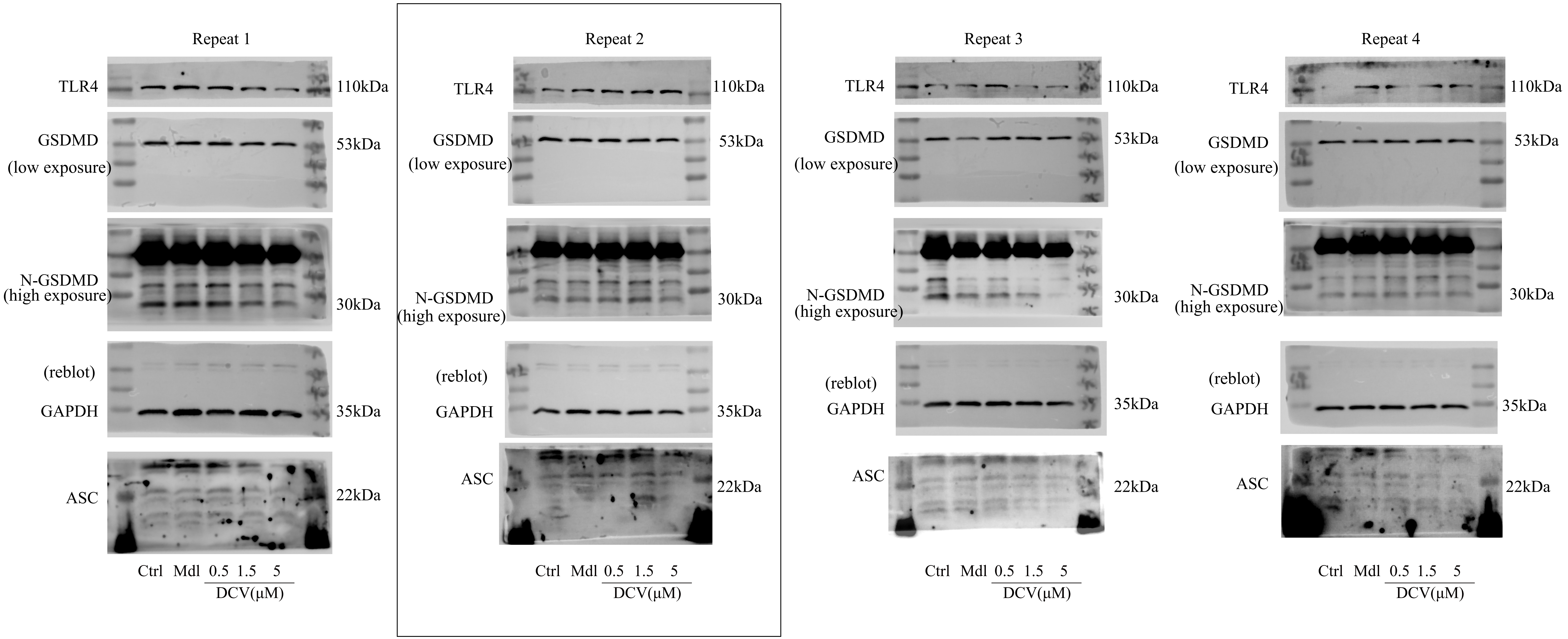

Fig. 3E-1

Original western blot for four repeats in Fig 3 (E, H)

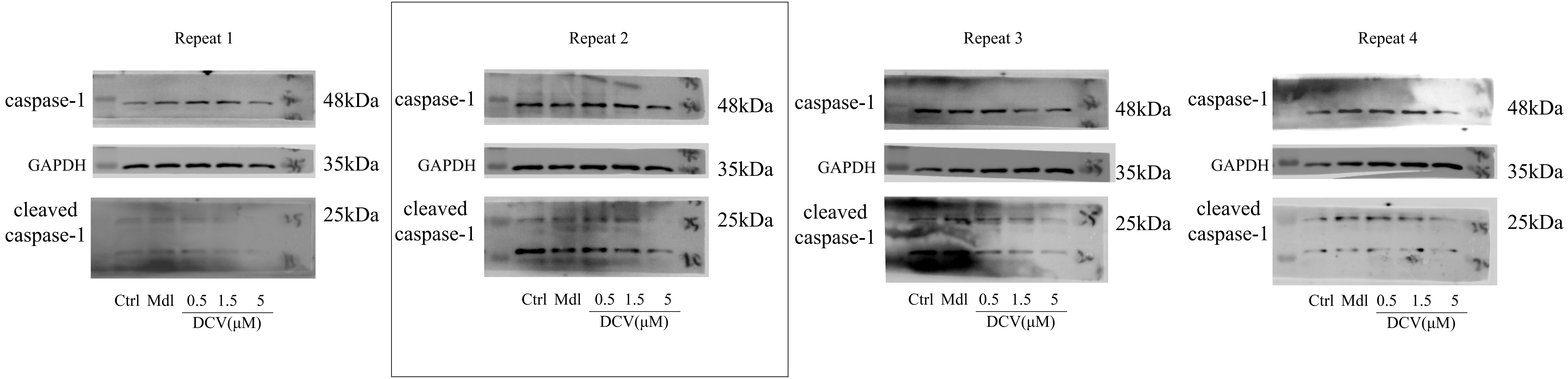

Fig. 3E-2

Original western blot for three repeats in Fig 3 (E, I, J)

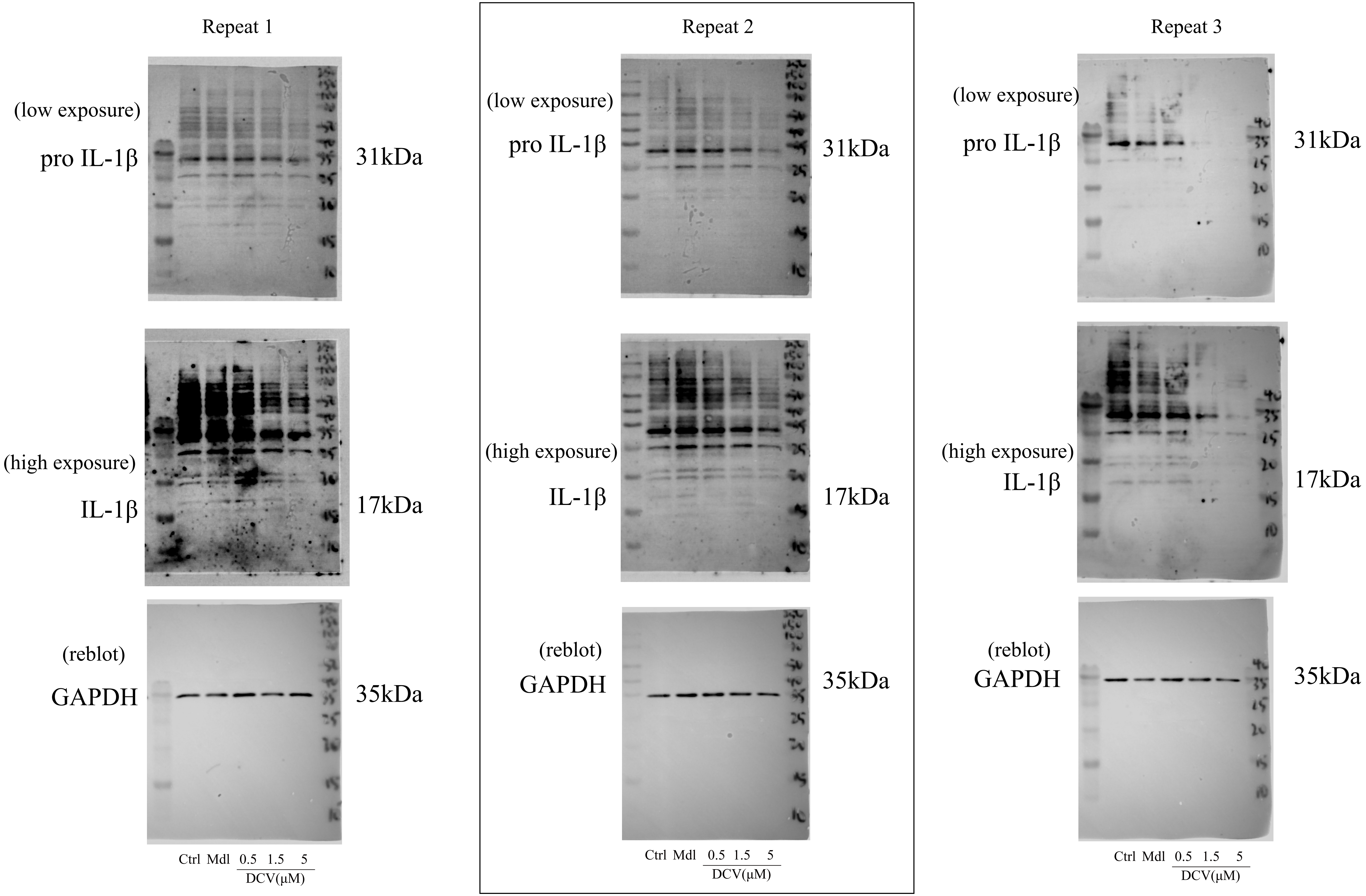

Fig. 3E-3
